# Rac1 Participates in Thermally Induced Alterations of the Cytoskeleton, Cell Morphology and Lipid Rafts, and Regulates the Expression of Heat Shock Proteins in B16F10 Melanoma Cells

**DOI:** 10.1371/journal.pone.0089136

**Published:** 2014-02-20

**Authors:** Burcin Gungor, Imre Gombos, Tim Crul, Ferhan Ayaydin, László Szabó, Zsolt Török, Lajos Mátés, László Vígh, Ibolya Horváth

**Affiliations:** 1 Institute of Biochemistry, Laboratory of Molecular Stress Biology, Biological Research Center, Hungarian Academy of Sciences, Szeged, Hungary; 2 Laboratory of Cellular Imaging, Biological Research Center, Hungarian Academy of Sciences, Szeged, Hungary; 3 Institute of Genetics, Laboratory of Cancer Genome Research, Biological Research Center, Hungarian Academy of Sciences, Szeged, Hungary; 4 Institute of Material and Environmental Chemistry, Department of Functional and Structural Materials, Research Center for Natural Sciences, Budapest, Hungary; Semmelweis University, Hungary

## Abstract

Eukaryotic cells exhibit a characteristic response to hyperthermic treatment, involving morphological and cytoskeletal alterations and the induction of heat shock protein synthesis. Small GTPases of the Ras superfamily are known to serve as molecular switches which mediate responses to extracellular stimuli. We addressed here how small GTPase Rac1 integrates signals from heat stress and simultaneously induces various cellular changes in mammalian cells. As evidence that Rac1 is implicated in the heat shock response, we first demonstrated that both mild (41.5°C) and severe (43°C) heat shock induced membrane translocation of Rac1. Following inhibition of the activation or palmitoylation of Rac1, the size of its plasma membrane-bound pool was significantly decreased while the heat shock-induced alterations in the cytoskeleton and cell morphology were prevented. We earlier documented that the size distribution pattern of cholesterol-rich rafts is temperature dependent and hypothesized that this is coupled to the triggering mechanism of stress sensing and signaling. Interestingly, when plasma membrane localization of Rac1 was inhibited, a different and temperature independent average domain size was detected. In addition, inhibition of the activation or palmitoylation of Rac1 resulted in a strongly decreased expression of the genes of major heat shock proteins *hsp25* and *hsp70* under both mild and severe heat stress conditions.

## Introduction

Although most cancer cells exhibit increased levels of the heat shock protein (HSP) subset of molecular chaperones [1], the exact mechanism of their elevated expression is still unresolved. One suggestion to explain the HSP overload typical of tumor cells is the “addiction to chaperones” hypothesis, which predicts that such an addiction is caused by the requirement for HSPs to chaperone the increased protein load that accompanies the transformation and the inherent instability of many mutant proteins [2]. Alterations of lipid profiles have been observed to be associated with various disease states, and it is suggested that changes in specific lipids are key factors in the onset and evolution of cancer [3]–[5]. The main difference between normal and tumor cell membrane resides in the status of the physical state and the nanoplatform (raft) organization [6]. This notion is well highlighted by the finding that changes in the ceramide level of cancer cells is a major factor controlling their apoptotic resistance [7].

The “membrane thermosensor” hypothesis postulates that heat stress can be sensed through subtle changes in the fluidity and microdomain hyperstructures of membranes influencing membrane-localized stress sensing and signaling and hence the expression of HSPs [4], [8], [9]. In the work reported here, we used the B16F10 mouse melanoma cell line, widely applied as a model system for the study of many aspects of cancer biology, including the heat shock response (HSR). The Rho family small GTPase Rac1 drives actin polymerization and is an important integrator of signals from integrins and growth factor receptors [10] and altered signaling is related to cell transformation, tumor invasion, and metastasis [11], [12]. Recently it was shown that there is a functional link between Rac1 and Hsp27 [13]. The dominant negative mutant of Rac1 (Rac1N17) attenuates paclitaxel-evoked apoptosis in melanoma M14 cells through upregulation of Hsp27, which inhibits the downstream drug-elicited caspase-3 activation [13]. However, how Rac1N17 regulates Hsp27 expression remains to be explored. It is known that actin filament reorganization is also involved in the process of apoptosis initiated by mild hyperthermia [14]. Destabilization of actin cytoskeletons proceeds with increasing stress temperature and leads to the active reorganization of the plasma membrane coincidental to heat-induced shrinkage and rounding of the cell shape [15]. Our earlier reasoning [16], [17] led us to assume that Rac1 also controls the HSP expression in B16F10 cells by acting as one of the key mediators of the stress-induced remodeling of surface membrane rafts.

In the present paper the involvement of Rac1 in mild and severe HSR was studied and among others it was shown that Rac1 takes part in the co-inducing activity of a drug used in ‘membrane lipid therapy’. Established Rac1-specific inhibitor NSC23766 and 2-bromopalmitate, which blocks the palmitoylation of Rac1 necessary for interaction with the liquid ordered membrane domains [18], [19], were investigated. Remarkably, administration of both agents caused a strongly reduced HSR. This altered HSR was apparently not correlated with a visible change in the level of HSF1 phosphorylation. We document here that the functionality of Rac1, and especially palmitoylation, markedly affects its thermally induced relocalization to the surface membrane. Moreover, the elevated association of Rac1 to the surface membranes can be linked causally to the earlier reported heat and membrane hyperfluidization-induced remodeling of cholesterol-enriched membrane microdomains [17], [20], [21].

## Materials and Methods

### Cell Culture

B16F10 mouse melanoma cell line (CRL-6475) was purchased from ATCC and was cultured in RPMI 1640 medium supplemented with 10% FCS and 4 mM L-glutamine. Mouse embryonic fibroblast (MEF) cells, a kind gift of Prof. Lea Sistonen [22], were grown in DMEM containing 10% FCS and 4 mM L-glutamine. Cells were maintained at 37°C in a humidified 5% CO_2_ atmosphere.

### Treatments and Reagents

For heat shock treatment, the plates were immersed for 1 h in a water bath set to the indicated temperature. In order to check the localization of Rac1 and F-actin proteins, HSF1 western blotting, mRNA levels of *hsp25* and *hsp70* genes, and alterations in membrane microdomains, samples were prepared immediately after heat shock treatment. The Rac1-specific inhibitor NSC23766 (NSC) (Santa Cruz Biotechnology) (100 µM) and the palmitoylation-inhibitor 2-bromopalmitate (2-Brp) (Sigma Aldrich) (25 µM) were applied for 2 h or 30 min, respectively, before heat treatment. These compounds were present in the medium during heat stress. The HSP co-inducer BGP-15 (10 µM) was added immediately before heat shock. To analyze HSP25 and HSP70 protein levels, fresh medium without any additional compound was added to the cells immediately after heat shock for an overnight recovery.

### Isolation of Crude Membrane Fraction for Testing Heat-induced Membrane Partitioning of Rac1

2×10^7^ B16F10 cells were used for each sample preparation. Cells heat-shocked for 1 h at the indicated temperatures (or kept at 37°C) were washed twice with ice-cold PBS, harvested by scraping on ice and resuspended in 1 ml of ice-cold SCA buffer (20 mM HEPES-KOH pH 7.5, 10 mM KCl, 1.5 mM MgCl_2_, 1 mM EDTA, 1 mM EGTA, 250 mM sucrose and protease inhibitor cocktail (Sigma)). Membrane was isolated by differential centrifugation as described by DerMardirossian *et al.* [23]. Membrane pellets were mixed with 1∶3 volumes of 3× Laemmli buffer, then assayed for total protein and equal amounts were analyzed by Western blotting. Membranes were probed with monoclonal Rac1 antibody (clone 23A8, Millipore) (1∶1000), then reprobed with Caveolin-1 antibody (C3237, Sigma Aldrich) (1∶1000). Expression levels were visualized with enhanced chemiluminescence (Amersham, USA). Band intensities were measured with Alpha View Software v.1.3.0.7. (Alpha Innotech, USA).

### Testing the Plasma Membrane/perimembrane Rac1 Localization by Confocal Microscopy

Following various pretreatments, the B16F10 cells were fixed with 4% paraformaldehyde (PFA), and then permeabilized with 0.1% Triton X-100. Rac1 antibody (1∶100) was used for labeling for 1 h at room temperature, which was followed by anti-mouse Alexa488 (A21202, Life Technologies) (1∶300) labeling for 1 h at 37°C. After washings, images were taken with a Leica SP5 AOBS confocal laser scanning microscope, using the 488 nm argon laser line for excitation and 500–530 nm spectral filter for emission detection. Images were analyzed with ImageJ software (http://rsbweb.nih.gov/ij/): whole cells and plasma membranes (PMs) were drawn around for at least 15 cells/3 views for each treatment. The average intensity of the pixels representing the PM was divided by the average intensity of the whole cell in an equatorial focal plane. The Student’s t-test was performed. Experiments were repeated 3 times; the results demonstrated the same tendency. A linear region of interest was chosen for every representative image. The fluorescence intensity of the pixels within this region was plotted.

### Membrane Microdomain Size Distribution Analysis

B16F10 cells grown in glass-bottom dishes were pretreated with 2-Brp for 30 min and then heat-shocked at 41.5°C or 43°C for 1 h or kept at 37°C. Immediately afterwards, the samples were washed and incubated with 1 µM BODIPY FL C12-sphingomyelin (Avanti) for 10 min at room temperature in order to label the raft domains. After washing the cells with RPMI without phenol red, images were taken with fluorescent microscope, applying the objective-based total internal reflection (TIR) configuration, using 488 nm light for excitation. Images were analyzed with CellProfiler (www.CellProfiler.org) and ImageJ software. The size (theoretical diameter) of each single detected microdomain was calculated as 2× * (Npix/π)–2, where X is the size of a pixel in nm and Npix is the number of pixels covering the actual domain. The domains found were sorted into classes, depending on the theoretical diameter. Calculations were carried out on at least 50 cells for each sample and the t-test was performed. Each experiment was repeated 3 times.

### Monitoring the Total Cellular F-actin Changes Induced by Heat Treatments in B16F10 Cells by Flow Cytometry

Following heat pretreatments (37.0, 41.5 or 43.0°C, 1 h), B16F10 cells were trypsinized, washed, collected and then fixed with 4% PFA, permeabilized with 0.1% Triton X-100 and labeled with Alexa Fluor 647 phalloidin (200 nM) (A22287, Life Technologies). Measurements were performed with a BD Accuri C 6 flow cytometer (BD Biosciences) with the 648 nm laser line for excitation and the FL4 channel for detection. Gating was based on the FSC/SSC and FL4A signals. The data were quantitated by using the CFLow Plus 1.0.227.2. software. Three independent experiments were performed.

### Quantitation of Surface Changes of B16F10 Cells Under Various Heat Stress Conditions and in the Presence of Rac1 Inhibitors with Scanning Electron Microscopy (SEM)

B16F10 cells were grown on sterile 5 mm cover slips in 24 well plates. After treatments, the cells on the cover slips were washed with PBS and fixed with 2.5% glutaraldehyde solution containing 4.5% glucose buffered with 75 mM cacodylate buffer (pH 7.2) for 3 h at room temperature. After 3 washes with 100 mM cacodylate buffer, secondary fixation was achieved by the addition of 1% osmium tetroxide buffered with 50 mM cacodylate (pH 7.2) for 3 h at room temperature. Subsequently, the cells were washed with distilled water, dehydrated in an ethanol series (25%, 50%, 75%, 90% and 100%) and then a 1∶1 mixture of ethanol/acetone, and subsequently kept in pure acetone. Finally, critical point drying was performed with CO_2_ in an E 3000 critical point drying apparatus (Quorum Technologies, Newhaven). The specimens were mounted on adhesive carbon discs and sputter-coated with gold in a SC7620 Sputter Coater (Quorum Technologies, Newhaven), and images were taken with an EVO40 scanning electron microscope (Carl Zeiss GmbH, Oberkochen) at 20.0 kV. Quantitation of the image surfaces was carried out by using ImageJ. Cell borders were drawn and pixel numbers within the borders were obtained and converted to µm^2^ units. For each sample, measurements were made on 30 cells, and t-tests were then performed.

### Confocal Laser Scanning Microscopy for Actin Imaging in MEF Cells Treated Under Various Heat Stress Conditions with the Rac1 Inhibitors NSC and 2-Brp

Confocal laser scanning microscopy was performed with a Leica SP5 AOBS confocal laser scanning microscope (Leica, Germany) on a DMI6000 microscope base. The microscope configuration: objective lenses: HC PL APO 20× (NA:0.7); HCX PL APO lambda blue 63× OIL (N.A: 1.40); sampling speed: 200–400 Hz; line averaging: 1–2×; pinhole: 1 airy unit; scanning mode: sequential unidirectional; excitation: 405 nm laser (DAPI), 633 nm HeNe laser (Alexa 647-phalloidin); spectral emission detectors: 417–525 nm (DAPI), 643–746 nm (Alexa 647-phalloidin). DAPI and Alexa 647-phalloidin images were pseudocolored blue and red, respectively. Composite images were prepared through the use of ImageJ software.

### qRT-PCR of *hsp25* and *hsp70* mRNA of B16F10 Cells Treated Under Various Heat Stress Conditions with the Rac1 Inhibitors NSC and 2-Brp

Immediately after heat shock or compound administration, cells were harvested in lysis buffer. Total RNA was isolated (NucleoSpin RNA II, Macherey-Nagel) and 1 µg of RNA was reverse-transcribed with RevertAid H Minus First Strand cDNA Synthesis Kit, (Fermentas). *hsp70 (Mm01159846_s1)*, *hsp25 (Mm00834384_g1)* and *gapdh (Mm99999915_g1)* primers with TaqMan probes (Applied Biosystems) and TaqMan Universal PCR Master Mix (Applied Biosystems) were used to prepare the reaction mixtures according to the instructions of the manufacturers. The PCR runs were performed on a BioRad-CFX96 instrument. Relative quantities of mRNAs were normalized to *gapdh*. Reported data are the means of the results of three independent experiments.

### Western Blotting

To analyze expression levels of Rac1, HSP25, HSP70 and the phosphorylation rate of HSF1, cells were harvested and lysed in Laemmli buffer. Equal amounts of proteins were loaded to 15% or 10% SDS gels depending on the size of the protein of interest, and western blotting was performed. Membranes were probed with monoclonal (mAb) Rac1, Caveolin-1, HSP25 (SPA 801, Stressgen), HSP70 (SPA 810, Stressgen), monoclonal HSF1 (Clones 4B4+10H4+10H8, Thermo Scientific), and Gapdh (Sigma-Aldrich) antibodies as indicated in the Figure legends. Expression levels were visualized with enhanced chemiluminescence. Band intensities were measured with Alpha View Software v.1.3.0.7. Reported data are the means of the results of three independent experiments.

## Results

### Heat Stress Triggers Accumulation of Rac1 on Membranes in B16F10 Cells

**Figure 1:**
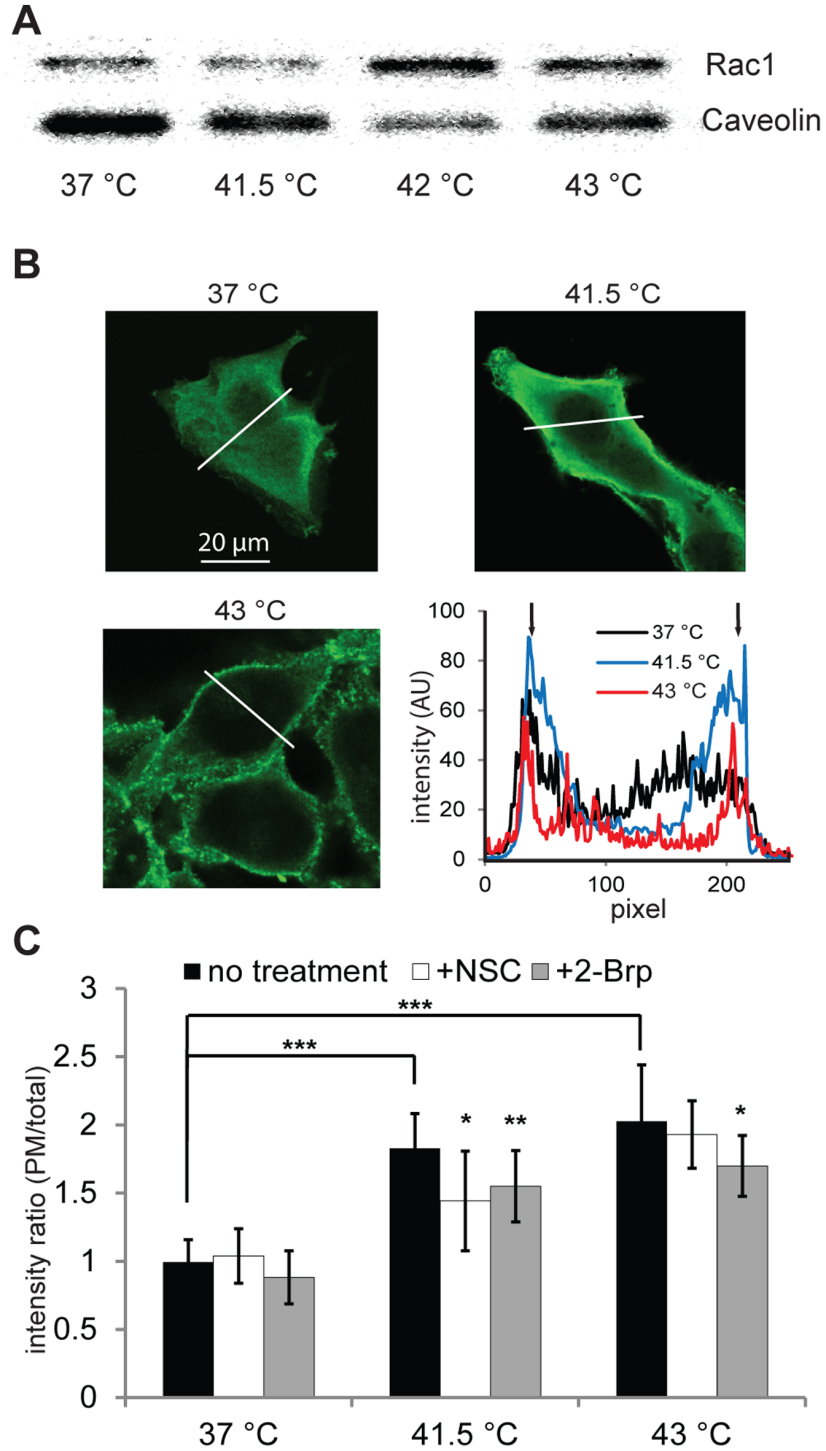
Translocation of Rac1 to membranes as a result of Rac1 inhibitor and heat shock treatment. (**A**) Localization of Rac1 to the crude membrane fraction in response to heat shock treatment. B16F10 cells were subjected to heat shock at the indicated temperatures for 1 h. Immediately after this, the crude membrane fraction was isolated and solubilized in Laemmli buffer. Equal amounts of proteins were run for western blotting. Rac1 probing was performed for the membrane, and caveolin immunostaining was used for normalization. Changes in normalized Rac1 band intensities: 37°C = 100, 41.5°C = 132, 42°C = 310, 43°C = 255. (**B**) Visualization of the association of Rac1 to the PM. B16F10 cells in glass-bottomed plates were kept in a water bath at the indicated temperatures for 1 h. The cells were then fixed, permeabilized and immunoreacted with Rac1 mAb, and probed with Alexa488-labeled secondary antibody by confocal microscopy. Intensity profiles of regions of interest on confocal images (indicated with white lines) are shown. Black arrows indicate PM. The red curve refers to 43°C, the blue curve to 41.5°C and the black curve to 37°C. (**C**) The effects of Rac1 inhibitor administration on the PM binding of Rac1 under heat stress conditions. Cells were treated/or not with the Rac1 inhibitor NSC or 2-Brp, heat-shocked/or not, and then immunostained as above. Confocal images were taken and 15 cells/3 views for each treatment were quantified by ImageJ. The bars represent the fluorescence intensity of the PM versus the fluorescence intensity of the whole cell. Black bars relate to cells not treated with inhibitor, white bars to NSC-treated and gray bars to 2-Brp-treated cells. Data are means ± SEM, n = 3, Student’s t-test was used for statistical analysis. *: p<0.05; **: p<0.01; ***: p<0.001.

It was earlier suggested that the small GTP-binding protein Rac1 may play a critical role in the early phase of heat stress management [11], [16]. Since translocation of Rac1 to the PM is assumed to be an essential step for the activation of downstream effectors [24] and the remodeling of the membrane microdomains [18], the effects of heat stress on the membrane fraction localization of Rac1 were first investigated on the highly metastatic B16F10 melanoma cells. From a comparison of the calculated relative band intensities of western blots (37°C = 100; 41.5°C = 132; 42°C = 310; 43.0°C = 255) the exposure of cells to 41.5°C did not significantly influence Rac1 membrane association (**Figure 1A**). In contrast, thermal treatment at 42 or 43°C led to a highly elevated association of Rac1 with cell membranes. Since Rac1 is also localized on endomembranes, such as mitochondria [25], where it is a binding partner of Bcl-2 and stabilizes its anti-apoptotic activity, we used confocal microscopy to demonstrate the heat stress-induced alterations in the PM localization of Rac1. Following thermal treatment (41.5°C or 43.0°C) Rac1 immunostaining was performed. The confocal microscopy results indicated that, in response to elevation of temperature to a clinically relevant range (41.5°C), Rac1 is directed to the PM (**Figure 1B,C**). Further increase of the temperature to 43.0°C did not give rise to any significant additional elevation in the PM-associated Rac1 pool. The small molecule NSC is a cell-permeable inhibitor of Rac1 which prevents its activation by Rac-specific guanine nucleotide exchange factors (GEFs) without affecting Cdc42 or RhoA activation [26]. It has been used in several cell lines to investigate the role of Rac1 in most diverse cellular functions [27]–[29]. Inhibition of Rac1 activity with NSC slightly but significantly (p<0.05) reduced the PM translocation of Rac1 under mild heat conditions (**Figure 1C**). Recent work identified the palmitoylation of Rac1 as a key mechanism controlling its membrane microdomain partitioning [18]. In response to the pretreatment of cells with 2-Brp, an effective and irreversible inhibitor of protein palmitoylation *in vivo* [30], we observed significant losses of both the mild (p<0.01) and the severe (p<0.05) heat-induced PM localization of Rac1 (**Figure 1C**). It should be noted that a small, but significant reduction in Rac1 PM localization was observed even in the control cells following 2-Brp administration, confirming the central role of palmitoylation in the subcellular compartmentalization of Rac1 [**18**].

### Palmitoylation of Rac1 Influences the Heat-induced Reorganization of PM Microdomains in B16F10 Melanoma:TIRF Microscopy Analysis

**Figure 2:**
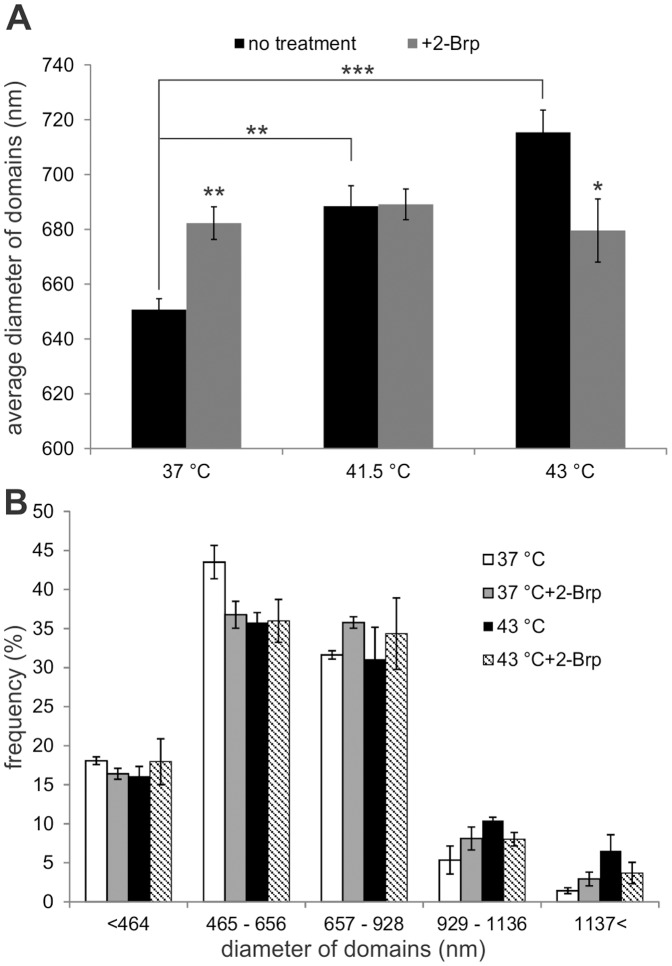
Change in size of PM microdomains as functions of temperature and 2-Brp treatment. (**A**) 2-Brp was added/or not to samples for 30 min, and they were then heat-treated at 41.5°C or 43°C or kept at 37°C for 1 h. After BODIPY FL C12-sphingomyelin labeling and TIR microscopy, the domain distribution was analyzed with ImageJ and CellProfiler software and the average domain sizes are shown. (**B**) From the experiment shown in (**A**), the PM microdomains were separated into five classes, depending on their sizes. The data shown are means ± SEM, n = 3.

To unravel the possible mechanisms underlying the capability of various membrane fluidizers for heat shock gene activation, we earlier documented that, apart from overall membrane hyperfluidization, a distinct reorganization of cholesterol- and sphingomyelin -rich microdomains (“rafts”) may also be required for the generation and transmission of stress signals to activate *hsp* genes in B16F10 cells [20]. We have also shown that such a reorganization of the lipid rafts must be a key determinant in the pleiotropic effects of hyperthermia, leading ultimately to cellular adaptation or lethality [21]. The Rac1 palmitoylation state has been shown to regulate both the fluidity and the domain organization of the PM [18]. Short exposure (30 min) of Cos-7 cells to 2-Brp, alters Rac1 subcellular compartmentalization [18] contrasting with other palmitoylated proteins, whose subcellular distribution is altered only after several hours [31], [32]. As stated by Navarro-Lérida *et al.* [18], this difference might reflect different rates of palmitate turnover. We tested the consequences of the palmitoylation inhibition using 2-Brp on the PM microdomain organization affected by heat treatment. The PM microdomain organization was measured by means of TIR microscopy using a BODIPY FL C12-sphingomyelin label on B16F10 cells. The domain size was analyzed with the freeware ImageJ software; domains were segmented with its fast Fourier transform (FFT) bandpass filter and CellProfiler. The sphingomyelin probe-labelled domains were separated into five classes, depending on their sizes (**Figure 2B**). The average diameter of the domains was clearly increased by a temperature elevation. However, 2-Brp pre-treated cells showed the same average domain size and size distribution independently of temperature. (**Figure 2** **A, B**). Interestingly, 2-Brp treatment resulted in a higher average domain size even at growth temperature. As was shown by Navarro-Lérida *et al.* [18], Cos7 cells expressing no Rac1 or a palmitoylation-deficient mutant have an increased content of disordered membrane domains. This is not the case in palmitoylation inhibitor treated B16F10 cells as the ratio of ordered-disordered domains was unchanged as a result of 2-Brp treatment (data not shown). A complete elucidation of the temperature independent effect of 2-Brp on lipid raft size and distribution awaits further studies and currently is in progress in our laboratory. The preparation of B16F10 Rac1 palmitoylation-incompetent mutant cells is at present under way through the use of the combined “Recombinase Mediated Cassette Exchange” (RMCE) and “Sleeping Beauty” (SB) gene transfer methods [33].

### Rac1 is Involved in Severe Heat Induced Changes in Cell Morphology

**Figure 3:**
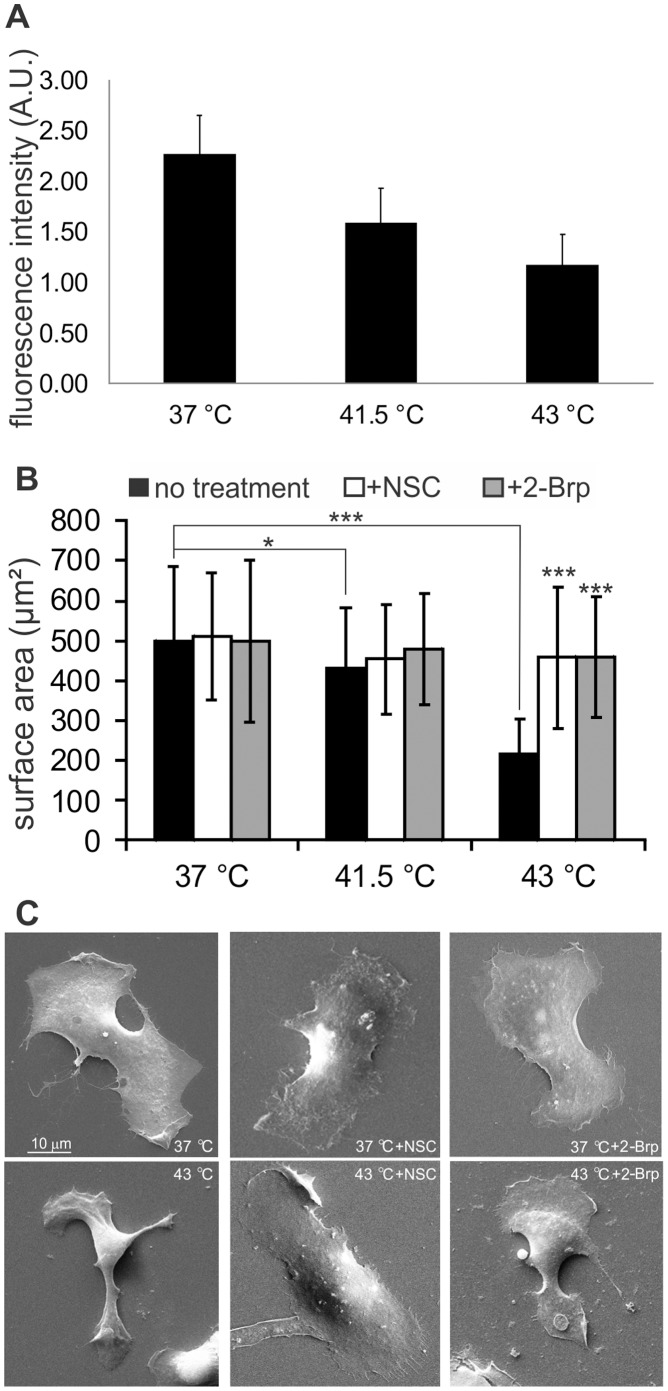
F-actin and cell morphology changes in B16F10 cells under heat shock conditions. (**A**) Measurement of heat stress-triggered F-actin alterations by using flow cytometry. After heat stress, cells were collected by trypsinization, fixed and labeled with Alexa 647 phalloidin. Flow cytometry measurements were made with a BD Accuri C 6 cytometer, and CFLow Plus 1.0.227.2. software was used for data analysis. (**B**) Surface area changes of B16F10 cells in response to heat shock and Rac1 inhibitor administration. B16F10 cells were subjected to stress conditions at 41.5°C or 43°C or kept at 37°C with or without inhibitor administration as indicated, and samples were fixed for SEM imaging. From the SEM images, 30 cells were chosen for the calculation of surface area with ImageJ software. Black bars relate to the lack of inhibitor treatment, while white and gray bars relate to NSC and 2-Brp administration, respectively. The data from three independent experiments are shown, along with the S.D. Student’s t-test was used for statistical analysis. *: p<0.05; **: p<0.01; ***: p<0.001. (**C**) Representative SEM photos of 37°C, 37°C+NSC, 37°C+2-Brp, 43°C, 43°C+NSC and 43°C+2-Brp-treated cells.

The active stress response of mammalian cells is classified as sequential and discontinuous heat-induced processes [34]–[36]. While the cell morphology, adhesion behavior and integrity of surface membranes have been shown to be unaffected by mild heat stress (40–41°C), the surface roughness of MX1 breast cancer cells increased as a consequence of the destabilization of the actin cytoskeleton [15]. For the monitoring of the heat-induced changes in the level of cytoskeletal F-actin following heat treatment (37.0, 41.5 or 43.0°C, 1 h), B16F10 cells were labeled with Alexa Fluor 647 conjugated to phalloidin. As evidence of the heat-induced destabilization of the F-actin network, flow cytometric investigation revealed a gradual but drastic reduction of F-actin levels in response to heat treatment (**Figure 3A**).

Earlier studies of the Rho family of GTP-binding proteins demonstrated that they play major roles in regulating the remodeling of the actin cytoskeleton, induced by stress and other extracellular signals. In fact, rearrangement of the actin filaments accompanied by striking changes in cell shapes were reported under comparatively mild heat stress conditions when MX1 breast cancer cells were used [15]. In order to follow the changes in the morphological features, we next applied SEM under conditions where the distinct reorganization of surface membrane microdomains has already been documented (see **Figure 2**). Calculated data of cell surface areas from SEM pictures indicated that heat treatment at 41.5°C did not cause pronounced changes in cell morphology (**Figure 3B**). Thermotreatment at 43.0°C resulted in heat-induced rounding of the cells (**Figure 3B**) leading to a significant reduction in the calculated cell surface area. When heat stress at 43.0°C was performed in the presence of either NSC or 2-Brp, this effect was completely absent (**Figure 3C**), clearly pointing to the key role of Rac1 in heat-induced changes in cell morphology.

### Rac1 is Involved in the Disorganization of Actin Filaments Upon Severe Heat Stress Treatment

**Figure 4:**
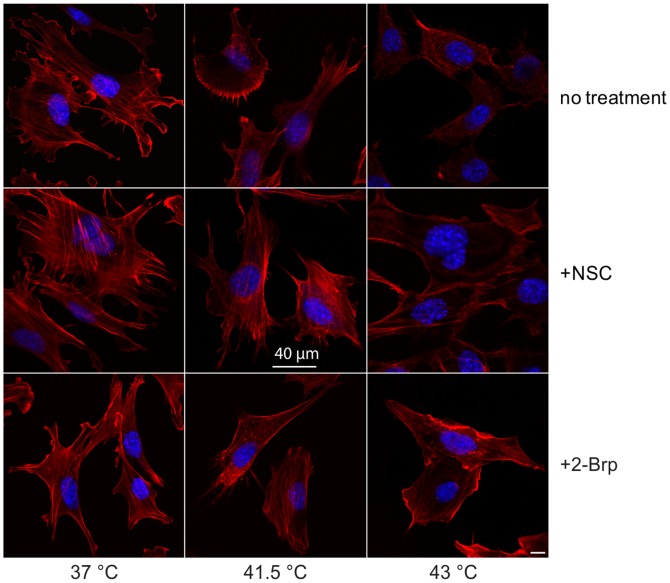
Effects of Rac1 inhibitors on the actin filaments of heat-and Rac1 inhibitor-treated MEF cells. Cells were subjected to selected heat stress conditions with or without NSC or 2-Brp treatment. After fixation and permeabilization, cells were labeled with Alexa 647-phalloidin (red, actin) and DAPI (blue, nuclei) and confocal microscopy was used for immunolocalization. Scale bar: 40 µm.

As discussed before, heat stress-induced changes in cell shape and cytoskeleton have been well documented through the use of various microscopy and imaging-based methods. Both microtubules and actin filaments are affected by heat stress in a variety of organisms [37]–[39]. Given the importance of Rac1 in the actin cytoskeletal organization, we used the Rac1-specific inhibitor NSC, a palmitoylation inhibitor (2-Brp) and confocal laser scanning microscopy to assess the potential action of Rac1 on the actin fiber organization during heat stress (**Figure 4**). Flat and well-spread fibroblasts are ideally suited for actin labeling and analysis, and MEF cells were therefore chosen for the experiments for the microscopic analysis of actin reorganization.

Cells with or without inhibitor pretreatement were subjected to either mild (41.5°C) or severe heat stress (43°C). Control cells were treated similarly, but kept at 37°C. Fixed cells were stained with fluorescent phalloidin to detect actin filaments. Mild heat stress did not cause any significant reorganization of the actin filaments in the untreated or the Rac1 inhibitor-treated cells. Actin-rich cell protrusions, several filopodia and lamellipodia were detected. On the other hand, the severe heat stress treatment at 43°C caused considerable fragmentation of the fine actin network in the untreated cells. Inhibition of Rac1 with NSC prevented this fragmentation considerably: long and intact actin fibers were still detectable in NSC-treated cells (**Figure 4**). Cell migration and spreading is driven by actin polymerization and actin stress fibers [40]. Control MEF cells exhibited a more rounded trapezoid-like cell morphology at 43°C, indicating reduced spreading behavior due to the disintegration of actin fibers at this temperature. The NSC-treated-cells, on the other hand, displayed a slender, elongated morphology resembling that of the control cells at 37°C (**Figure 4**).

Rac1 is known to be modified by palmitoylation at the carboxy terminal. This modification targets Rac1 for stabilization in the actin cytoskeleton-linked ordered membrane regions at the PM [18]. Using the palmitoylation inhibitor 2-Brp, we tested the actin morphology of MEF cells following mild and severe heat stress treatment. Elongated cells with actin-rich protrusions were often detected in 2-Brp-treated cells at 41.5°C. At 43°C, the inhibition of palmitoylation resulted in better-preserved, intact actin fibers and elongated actin-rich protrusions. Similarly to the NSC-treated cells, only a moderate actin depolymerization was detected (**Figure 4**).

### Rac1 Takes Part in the Heat İnducibility of *hsp25* and *hsp70*

**Figure 5:**
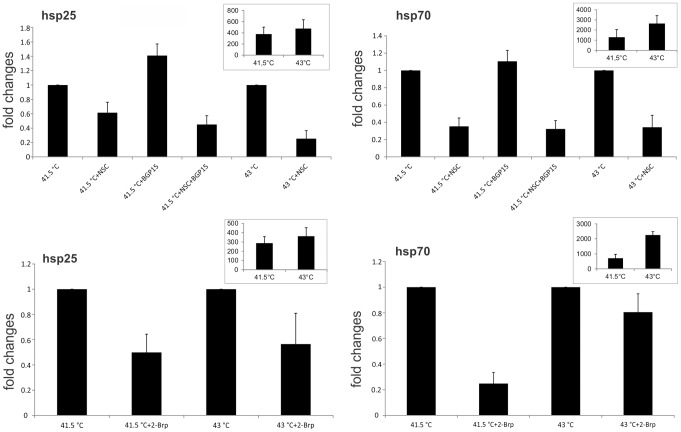
Effects of NSC and 2-Brp on *hsp25* and *hsp70* gene expressions under heat shock conditions. B16F10 cells were either treated/or not with NSC or 2-Brp for 2 h or 30 min, respectively, and then exposed/or not to the indicated heat shock temperatures for 1 h. Total RNA was isolated immediately after the heat shock, and the expression levels of *hsp70* and *hsp25* mRNAs were measured by quantitative RT-PCR and normalized to *gapdh*. If BGP-15 (HSP co-inducer) was applied, it was administered at 10 µM during heat shock. Data shown are fold changes compared to the mRNA levels measured in inhibitor untreated, heat shocked samples which was considered 1. Inserted graphs represent the fold changes compared to the mRNA levels measured in 37°C samples. Data are means ± SEM, n = 3.

In previous studies we have shown the elevated gene expression levels of *hsp25* and *hsp70* under stress conditions in B16F10 cells [4], [20], [41]. In order to investigate the possible role of Rac1 in the HSR, the effects of the Rac1 inhibitor NSC and 2-Brp were tested by using quantitative RT-PCR. The administration of NSC decreased the mRNA levels of both *hsp25* and *hsp70* under mild (41.5°C) heat shock. Moreover the *hsp* co-inducing activity of BGP-15 was also inhibited [17], [42]. Under severe heat shock conditions (43°C), both mRNA levels were drastically inhibited by the Rac1 inhibitor NSC. In cells heat-treated at 41.5°C, the administration of 2-Brp resulted in an inhibitory effect on the induction of both *hsp25* and *hsp70* mRNA, though the inhibition was more pronounced for *hsp70*. However, under severe heat stress (43°C), the palmitoylation inhibitor affected the *hsp25 mRNA* far more than the *hsp70* mRNA inducibility (**Figure 5**).

### Rac1 Inhibitor NSC Attenuates the HSR at Protein Level

**Figure 6:**
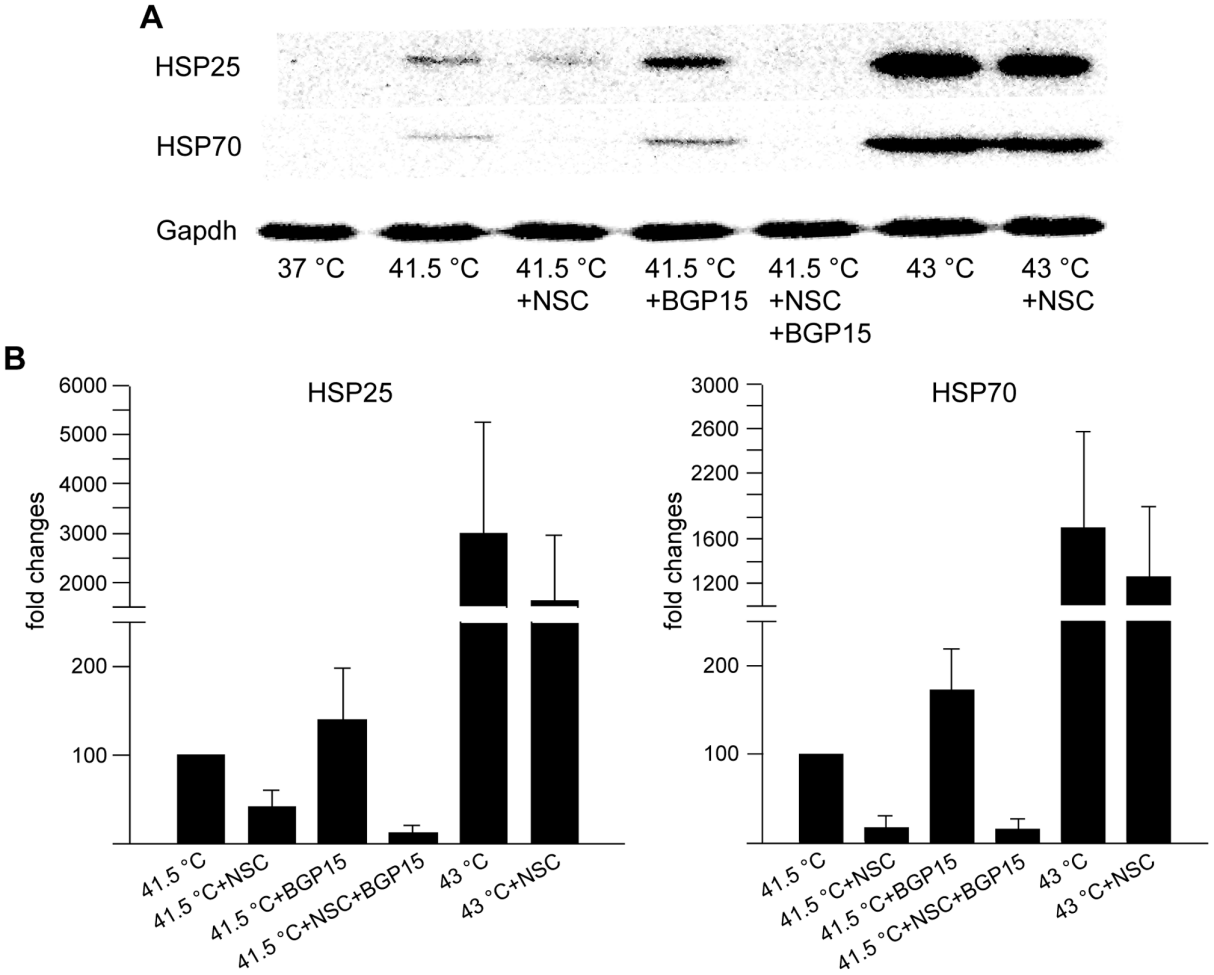
Effects of NSC and 2-Brp on HSP25 and HSP70 protein levels under heat shock conditions. Cells were treated as described for **Figure 5** and were collected after an overnight recovery at 37°C. (**A**) Western blotting was performed for equal amounts of total protein samples. Membranes were probed with anti-HSP25, anti-HSP70 and anti-Gapdh antibodies. (**B**) The band intensities of protein expression levels were quantitated and normalized to Gapdh. In calculations of fold changes, the protein levels of 41.5°C samples were taken as 100. Data are means ± SD, n = 3.

In order to confirm the changes in HSR following the inhibition of Rac1 activity and membrane association, the levels of the proteins HSP25 and HSP70 were measured by western blotting. B16F10 cells were exposed to compound administration and stress conditions as indicated in **Figure 6A**. The elevated expression of HSP25 and HSP70 induced by either mild or severe heat stress and co-induction of the HSR at 41.5°C with the hydroximic acid BGP-15 was markedly inhibited by the specific Rac1 inhibitor NSC. Importantly, this effect was more pronounced under mild heat shock conditions (**Figure 6A,B**).

### Rac1 İnhibition does not Affect the Level of HSF1 Phosphorylation

**Figure 7:**
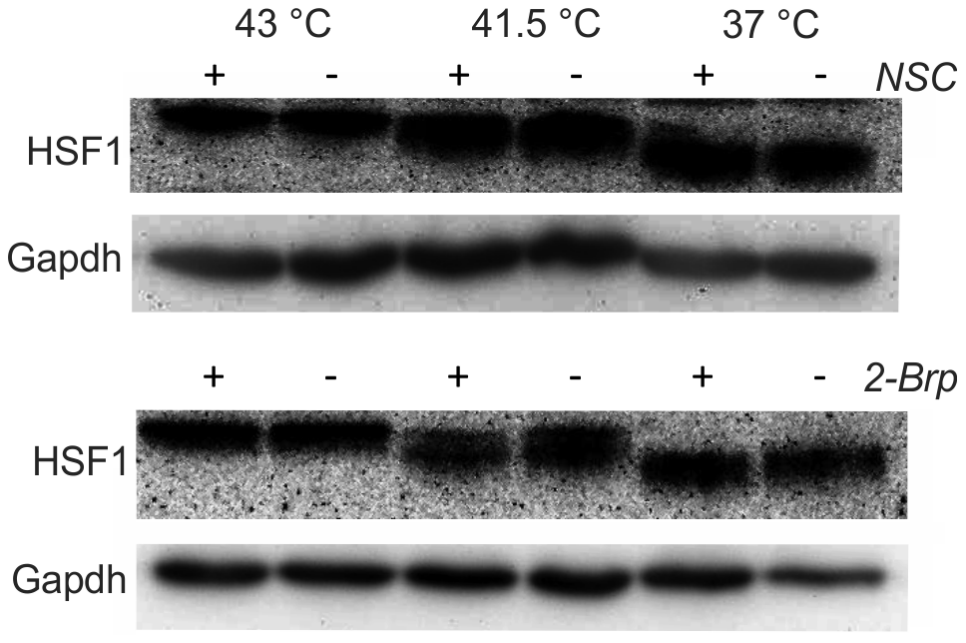
Effect of Rac1 inhibition on hyperphosphorylation level of HSF1. B16F10 cells were treated or not with inhibitors (NSC or 2-Brp) prior to the indicated heat shock conditions. Immediately afterwards, samples were harvested and equal amounts were used for western blotting. Membranes were probed with anti-HSF1 and anti-Gapdh antibodies.

The expression of the HSPs is mediated through activation of the heat shock transcription factors, of which HSF1 is the master regulator in vertebrates [43]. Under stress conditions, HSF1 homotrimerizes, localizes to the nucleus, becomes transcriptionally competent and is hyperphosphorylated. We demonstrated here that the extent of HSF1 hyperphosphorylation was indeed proportional to the severity of the heat shock, but neither NSC nor 2-Brp modulated the heat-induced level of HSF1 phosphorylation (**Figure 7**). Since the levels of the transcription of both *hsp25* and *hsp70* were equally and strongly affected by the Rac1 inhibitors that we applied, it is feasible that such a Rac1- mediated effect on heat shock gene transcription does not involve change in the net phosphorylation of HSF1. In fact, the DNA-binding and transactivation capacity of HSF1 is coordinately regulated through multiple posttranslational modulations (e.g. acetylation), protein–protein interactions and subcellular localization [43]. Further, heat shock gene expression is thought to be controlled at the level of promoter clearance [44]. Accordingly, the expression of heat shock genes should be particularly sensitive to the cellular level of Pol II elongation factors. A variety of Pol II elongation factors are recruited to sites of heat shock gene expression during heat shock [45]. However, the signalling pathways responsible for RNA polymerase activation remain largely unclear.

## Discussion

Since a small GTPase Rac1 has been suggested to participate in the cellular response to various kinds of stresses, we examined whether Rac1 is involved in several features of the HSR under mild (41.5°C) and severe (43°C) heat shock conditions. It was earlier assumed that an increase in the intracellular level of calcium, well documented under heat stress conditions in our laboratory [46], is the major regulator of the activation and membrane translocation of Rac1 [47]. Among others, the initiation of Rac1 signaling requires GTP loading and a posttranslational modification, i.e. palmitoylation [18]. Palmitoylation targets Rac1 for stabilization of the actin cytoskeleton-linked cholesterol-rich liquid-ordered (Lo) PM microdomains. We have demonstrated here that both moderate and severe heat shock induce the membrane translocation of Rac1 and that the translocation could be partially blocked by using the selective Rac1 activity inhibitor NSC and the palmitoylation inhibitor 2-Brp.

By monitoring the patterns of surface membrane microdomains by confocal and ultrasensitive single molecular microscopy, we established a phenomenological relationship between the specific distribution of lipid nanostructures (“rafts”) and the concomitant changes in the levels of HSPs [17], [20], [48], [49]. Here we showed that a decrease in the percentage of larger PM microdomains (929–1136 nm and 1137< nm) at the expense of their smaller counterparts is paralleled by a reduction in HSR. We also documented that, under conditions when the PM localization of Rac1 is inhibited by 2-Brp administration, there is a noteworthy decrease in raft domain size. Taken together, our findings further corroborate the hypothesis that the level and size distribution pattern of lipid rafts very likely participate in the early steps of the mechanism of raft-associated stress sensing and signaling [3]. The lipid raft hypothesis postulates that primarily the selective interactions involving sphingolipids, cholesterol and membrane proteins contribute to lateral membrane heterogeneity [50]. Although the raft concept of membrane organization has been subject to controversy, the model is now gaining increased acceptance through studies applying novel methodologies [51], [52]. These metastable membrane assemblies can be stabilized locally by lipid–lipid and lipid–protein interactions to coalesce and form functional domains for signal transduction. The mechanisms that govern such associations in live cell membranes during heat stress remain unclear [53].

Both microtubules and actin filaments are known to be affected by heat stress in a variety of organisms, and Rac1 has been shown to play important roles in cytoskeletal organization. Indeed, both the Rac1 inhibitor NSC and the palmitoylation inhibitor 2-Brp prevented the severe heat stress-induced changes in cell shape and cytoskeleton, as documented with the use of confocal microscopy and SEM imaging-based methods. In order to make a more profound assessment of the potential role of Rac1 on actin fiber organization during heat stress, we applied confocal laser scanning microscopy, using the flat and well-spread MEF fibroblast model. Thermal treatment at 43.0°C led to heat-induced rearrangements of actin filaments and shrinkage and rounding of the cells, which resulted in a significant reduction in the calculated cell surface areas. When severe heat stress was applied in the presence of either NSC or 2-Brp, this effect was completely absent, clearly pointing to the central role of Rac1 in the heat-induced changes in cell morphology.

As a consequence of the inhibition of either the activity or the PM localization of Rac1, we observed a clear parallel decrease in *hsp25* mRNA level under either mild or severe heat stress conditions. It was noteworthy that, whereas both Rac1 inhibitors caused a sizable decrease in the formation of *hsp70* mRNA at 41.5°C, in contrast with NSC, 2-Brp remained apparently ineffective under severe heat stress conditions.

It is to be noted that the present study provided full support for the finding that the HSP co-inducer BGP-15 enhanced activation of HSP expression involves Rac1 signaling [17]. As noted earlier, since we are fully aware of the nonspecific and pleiotropic effect of 2-Brp, further studies are currently under way in our laboratory with palmitoylation-incompetent, constitutively active and dominant negative Rac1 mutant B16F10 cells.

Finally, as we discussed above, neither NSC nor 2-Brp appeared to affect the detectable level of HSF1 phosphorylation. It should be noted, however, that in their early studies of the role of Rac1 in JNK activation and HSF1, Han *et al*. [11] concluded that, although a specific signal pathway involving Rac1 is probably linked to heat shock-induced HSF activation and HSP expression, a Rac1-independent mechanism is also involved, especially in the severe HSR. Rac1 activation may therefore be necessary, but not sufficient, for the thermally induced activation of HSF1 [11].

Although the mode of HSF1 activation in response to various stresses is likely to follow the same principle, there are stimulus-specific differences, arguing against a single common signal pathway to activate HSF1. HSF1 itself could act as a hub for stress-induced gene activation, providing a relay point for the downstream signaling of different stress stimuli [54]. As suggested by Akerfelt *et al.* [43], the activation and attenuation mechanisms of HSFs require additional mechanistic insight. The roles of the multiple signal transduction pathways involved in the post-translational regulation of HSFs are only now being discovered and are clearly more complex than anticipated.

Finally, the cellular stress management can only be perceived as a network of pathways with plasticity, feedback loops and crosstalks that ensure the ultimate fitness of the cells. An understanding of the of importance of concerted action of membranes, lipids, actin cytoskeleton, and the membrane interacting stress protein molecular chaperones in heat sensing and signaling and the acquisition of thermotolerance following heat priming is inevitable.
